# Anti-Obesity Medication Use in Children and Adolescents with Prader–Willi Syndrome: Case Review and Literature Search

**DOI:** 10.3390/jcm10194540

**Published:** 2021-09-30

**Authors:** Victoria E. Goldman, Monica N. Naguib, Alaina P. Vidmar

**Affiliations:** 1 Department of Pediatrics, Children’s Hospital Los Angeles, Los Angeles, CA 90027, USA; vigoldman@chla.usc.edu; 2 Center for Endocrinology, Children’s Hospital Los Angeles, Los Angeles, CA 90027, USA; mnaguib@chla.usc.edu; 3 Department of Pediatrics, Keck School of Medicine of University of Southern California, Los Angeles, CA 90033, USA

**Keywords:** Prader-Willi syndrome, anti-obesity medication, topiramate, metformin, oxytocin, semaglutide, liraglutide, naltrexone-bupropion

## Abstract

(1) Background: children with Prader-Willi syndrome (PWS) have high obesity rates due to hyperphagia and decreased metabolic rates. Although anti-obesity medications (AOMs) are prescribed to this population, there are no consensus guidelines on acceptability, safety, and efficacy. We present literature review and case series on AOMs in youth with PWS. (2) Methods: we performed PubMed review from January 2000 to April 2021 utilizing keywords: “Prader-Willi syndrome” or “PWS” and “medication” including: topiramate, metformin, phentermine, liraglutide, orlistat, oxytocin, semaglutide, naltrexone-bupropion. For our case series, patients were identified through retrospective chart reviews from a multi-disciplinary PWS clinic. Eligibility criteria: age ≤ 18 years, genetically confirmed PWS, AOM use for at least 16 weeks, and recent anthropometric data. (3) Results: a literature search yielded 14 articles (3 topiramate, 1 metformin, 4 liraglutide, 5 oxytocin, 1 naltrexone–bupropion). All studies reported improved hyperphagia with variable BMI effects. Ten adolescents met case series eligibility (mean age 13.2 ± 2.6 years, 40% female; AOMs: 6 metformin, 5 topiramate, 2 semaglutide, 3 liraglutide). After AOM course, 60% had decreased or stable BMI z-score. No significant side effects. (4) Conclusions: results suggest AOMs may be useful for weight management in youth with PWS. Additional studies are required to validate findings and support AOM treatment guidelines.

## 1. Introduction

Prader-Willi syndrome (PWS) is a genetic disorder with a predominant symptom of hyperphagia leading to severe obesity with numerous subsequent life-limiting, obesity-related comorbidities [1]. PWS is caused by an alteration to chromosome 15 (15 q11–q13) due to parental deletion of the region in 65–75% of cases, maternal uniparental disomy in 20–30%, or an imprinting defect in 1–3% [2]. It is the most common syndrome leading to obesity, with prevalence estimated at 1 in 10,000 to 1 in 30,000 [1,3]. There are many contributing factors to the severe obesity phenotype: hypothalamic abnormalities of satiety control resulting in hyperphagia, disruption in hormones regulating food intake, delayed gastric emptying, and reduced energy expenditure because of hypotonia, growth hormone deficiency, sleep disorders with reduced REM, and central as well as obstructive apnea [1,4]. Individuals with PWS have dysregulation at the level of the hypothalamus of Neuropeptide Y (NPY), agouti related protein (AgRP), and gamma-aminobutyric acid (GABA) neurons, which appear to be associated with the deletion of SNORD116 in the PWS critical region resulting in hyperphagia [1,3]. In addition, individuals with PWS have increased ghrelin levels with lower insulin and peptide YY (PYY) levels with associated leptin and insulin resistance at the blood-brain barrier, which decreases the hormones that promote satiety at the level of the hypothalamus [1,3]. 

Current treatments for obesity in PWS include growth hormone, to increase muscle mass while decreasing fat mass, strict supervision of food intake, caloric restriction, and oxytocin for improved infant feeding [2]. Studies on anti-obesity medications (AOMs) have been rising, but are still limited in pediatric populations, especially subgroups, such as patients with PWS [5]. There are currently no consensus guidelines on the use of AOMs in PWS. However, there are a number of AOMs currently used in varied populations that could yield promising results in patients with PWS. The medications we focused on include topiramate, metformin, phentermine, liraglutide, orlistat, oxytocin, semaglutide, and naltrexone-bupropion. 

Given the research gap of anti-obesity medications in youth with PWS, we conducted a literature review on PWS and AOM use in children and adolescents. Additionally, we completed a case series of pediatric patients with PWS on AOMs from a multi-disciplinary PWS clinic at a single, urban, diverse children’s hospital. We present a literature review of 81 articles, of which 14 met inclusion criteria, as well as a retrospective case series of 10 patients with PWS who completed, at minimum, a 12-week course of AOMs for the indication of weight management. The objective of this study is to describe the impact of AOMs on the body mass index (BMI) status and eating behaviors of patients with PWS, and catalog the side effect profiles of each medication. 

## 2. Materials and Methods

A comprehensive literature review was conducted of the PubMed database, with a primary objective of identifying documented reports of the use of AOMs (including: topiramate (Johnson & Johnson subsidiary Janssen Pharmaceuticals, Raritan, NJ, USA), metformin (Bristol-Myers Squibb, New York, NY, USA), phentermine (KVK Tech, Newtown, PA, USA), liraglutide (Novo Nordisk, Plainsboro, NJ, USA), semaglutide (Novo Nordisk Inc., Plainsboro, NJ, USA), orlistat (GlaxoSmithKline, Philadelphia Navy Yard, PA, USA), oxytocin (Novartis Pharma, Basel, Switzerland), and naltrexone–bupropion (Currax Pharmaceuticals, Brentwood, TN, USA)) in youth with PWS. There are several medications under investigation, in early phase clinical trials, including diazoxide choline, setmelanotide, livoletide, and tesofensine with metoprolol, which were not included in this review [6,7,8]. The search was conducted in April 2021 and included date limits of January 2000 until April 2021. The terms used were “PWS” or “Prader Willi Syndrome” and “*medication*.” Medications included “topiramate, metformin, phentermine, liraglutide, orlistat, oxytocin, semaglutide, naltrexone bupropion.” Results were screened to include trials, studies, and case reports on individuals (under age 21) with PWS who were taking one of the aforementioned AOMs. There were 81 results with 3 duplicates. 78 abstracts were reviewed and 64 were excluded for not meeting full criteria. The remaining 14 results were formally evaluated on review of the full-text article. All identified articles were read in full, with relevant information extracted and summarized (Table 1).

A retrospective chart review of a single, urban, tertiary care multi-disciplinary PWS clinic database was conducted for all patients with PWS cared for from July 2018 to June 2021. Ethical approval for this study was gained through the Children’s Hospital Los Angeles Institutional Review Board. Informed consent was obtained from all patients and their parents. Eligibility criteria included: (1) age 18 or younger; (2) genetic confirmation of PWS; (3) anthropometric measurements within the last 12 months; (4) BMI greater than or equal to the 95th percentile; (5) prescription for AOM; and (6) confirmation of AOM use for at least 12 weeks. The results are summarized descriptively as a case series. BMI z-score was utilized in our analysis and reporting to account for age and sex given all patients were at different stages of puberty with varying height velocities. A total of ten patients met the inclusion criteria and were included in the case series (Table 2).

## 3. Results

### 3.1. Summary of Literature Review

The literature review of PWS and AOMs using PubMed from January 2000 to April 2021 yielded 14 results. Articles were excluded for the following reasons: different language (*n* = 2), wrong populations (*n* = 6), adults only (*n* = 4), those with type 2 diabetes (*n* = 8), no focus on AOM use (*n* = 12), animal studies (*n* = 17), review articles (*n* = 9). There were 14 studies that met criteria, including 3 studies on topiramate, 1 on metformin, 4 on liraglutide, 5 on oxytocin, 1 on naltrexone-bupropion (Table 1). There were 0 studies for phentermine, orlistat, and semaglutide. The included studies were: 6 case reports, 6 double-blind placebo-controlled studies, 1 open label study, and 1 pilot study. Ages in the studies ranged from 3 to 45 years. Sample sizes ranged from 1 to 62 individuals. All studies recruited females and males. Results focused on changes in weight and BMI, hemoglobin A1c (HbA1c), mood and behavior, and adverse events.

#### 3.1.1. Topiramate 

Topiramate is a medication that blocks sodium and calcium channels, inhibiting the excitatory glutamate pathway and subsequently enhancing the inhibitory effects of GABA. It is commonly thought of as a medication for migraines and seizures, with off-label use for binge eating disorder, essential tremor, mood stabilization, and weight loss [9]. The mechanism for weight loss is not fully understood, but may be due to: inhibitory effects on glutamate that increase food intake through hypothalamus stimulation, effects on GABA and energy expenditure, or decreases in NPY that have been shown to stimulate appetite [10]. Multiple clinical trials demonstrate associations between topiramate and weight loss in the general population. A meta-analysis of 3320 individuals in the general population showed an average weight loss of additional 5.34 kg compared with placebo [11]. The most common side effects include fatigue, dizziness, and mood changes [10]. Topiramate is not currently Food and Drug Administration (FDA)-approved for obesity management. 

Literature review using “PWS” or “Prader Willi Syndrome” and “Topiramate” from January 2000 to April 2021 demonstrated 10 results, 3 of which reported the effects of starting patients with PWS on AOMs. A 2019 double-blind randomized placebo-controlled study reported a trend in decreased BMI without statistical significance, as well as a dose-dependent improvement in hyperphagia among 62 patients (ages 12–45 years) over eight weeks [12]. A 2003 open label study demonstrated weight loss or decreased weight gain and improved mood in seven patients with PWS [13]. Lastly, a 2018 case report described an eleven-year-old boy with PWS who had reduced aggression and “demand for food” following initiation of topiramate [14]. The remaining articles focused on adults, psychiatric symptoms, or were in a language other than English. 

#### 3.1.2. Metformin 

Metformin is classically used in type 2 diabetes for glycemic control without weight gain and with possible weight loss [15]. Metformin’s mechanism of action leading to weight loss and changes in appetite is multi-factorial, including decreased glucose production in the liver and glucose absorption in the intestine, as well as increased insulin sensitivity [4]. By increasing insulin sensitivity at the blood-brain barrier, more insulin may reach hypothalamic receptors, contributing to increased satiety [4]. Metformin also increases glucagon-like peptide-1, PYY, and lactate production, which can all contribute to decreased appetite [16,17]. Additionally, metformin helps increase catabolic pathways through AMP-activated protein kinase stimulation [16]. A 2018 systematic review of metformin’s effect on weight reduction in children and adults showed variable effects, with smaller reductions in children [18]. The side-effect profile includes abdominal discomfort with diarrhea, nausea, and vomiting. Rarely, lactic acidosis can occur [15,16,17]. Metformin is not currently FDA-approved for obesity management. 

There were 15 results for PWS and metformin. A 2014 pilot study examined 21 children with PWS and 6 with early morbid obesity, who were treated with metformin and demonstrated an improvement in food-related distress, anxiety, and increased satiety, but no significant weight loss in PWS [4]. Three studies included in the search review metformin with liraglutide, and will be discussed in the “Liraglutide” section based on primary focus. Excluded studies included articles with incorrect patient populations. 

#### 3.1.3. Phentermine 

Phentermine is a sympathomimetic medication predominantly used for obesity that is thought to release norepinephrine in the hypothalamus, thereby reducing hunger [19]. It is also thought to increase serotonin and dopamine reuptake [19]. Side effects include dizziness, dry mouth, gastrointestinal symptoms, tachycardia, hypertension, and difficulty sleeping [19]. Phentermine is currently FDA-approved for obesity management in patients 16 years and older. There were 0 results when searching for PWS and phentermine.

#### 3.1.4. Glucagon-Like Peptide-1 (GLP-1) Receptor Agonist 

Glucagon-like peptide-1 receptor agonist stimulates insulin release, inhibits glucagon secretion, slows gastric emptying, and increases satiety after eating [20,21,22]. In addition, there is a reduction in ghrelin, which is elevated in PWS [21]. Side effects are predominately gastrointestinal [23]. Liraglutide is the short acting daily preparation and semaglutide is the depot version that is administered once weekly. There have been limited studies of the weight loss effects of GLP-1 receptor agonists in pediatric populations [23]. Liraglutide is FDA-approved for obesity management for patients 12 years and older. There were six results for PWS and liraglutide, with four case reports meeting inclusion criteria. These included: a 17-year-old with PWS on liraglutide and metformin who had weight loss, increased satiety, as well as lower HbA1c and fasting glucose; an 18-year-old with PWS and dyspnea requiring mechanical ventilation due to severe obesity who maintained weight reduction on liraglutide; a 13-year-old with PWS and diabetes, with a decrease in glucose and HbA1c on liraglutide and empagliflozin; and a 19-year-old with PWS and diabetes who had improved glycemic control, HbA1c, insulin resistance, and weight loss on liraglutide and empagliflozin [21,24,25,26,27]. The remaining cases were focused on populations over the age limit or in a different language. The most common side effects include gastrointestinal symptoms [27,28]. Semaglutide is FDA approved for obesity management. There were 0 results when searching for PWS and semaglutide. 

#### 3.1.5. Orlistat 

Orlistat is a medication specifically aimed at obesity management. It is a lipase inhibitor that inhibits gastric and pancreatic lipases, which typically break down and digest dietary fat into absorbable free fatty acids and monoglycerides [29]. A systematic review reported three studies focused on orlistat in pediatric populations that demonstrated greater BMI reductions compared to placebo [30]. Gastrointestinal symptoms are the predominant side effects [29]. Orlistat is FDA-approved for obesity management for those 12 years and older. The one result from literature review was excluded for wrong focus.

#### 3.1.6. Oxytocin 

Oxytocin has a variety of uses, primarily for obstetric and gynecologic reasons through activation of G-protein-coupled receptors and increased prostaglandin production. However, it has also been found to have weight loss potential through increasing energy expenditure and lipolysis, as well as reducing appetite leading to decreased food intake [31,32,33]. Side effects include increased heart rate and gastrointestinal symptoms [33]. Oxytocin is FDA-approved for obesity management for those 12 years and older.

Oxytocin provided the most results with 46 articles. There were five studies on the administration of intranasal oxytocin in children with PWS that met inclusion criteria. Three studies showed improvement in social and food-related behaviors [34,35,36]. Two studies showed limited positive effects [37,38]. Excluded studies included animal studies, wrong medications or patient populations, alternative focus, and reviews.

#### 3.1.7. Naltrexone-Bupropion

Naltrexone-bupropion is a combination medication used to treat obesity and impulsive behavior. Naltrexone is an opioid receptor antagonist used as monotherapy for alcohol and opioid dependence and bupropion is a dopamine/norepinephrine reuptake inhibitor used as monotherapy to treat depression and smoking [39]. The combination medication works through the anorexigenic effect of a-melanocyte-stimulating hormone from pro-opiomelanocortin neurons of the hypothalamus [39,40]. Side effects include increased blood pressure, headache, insomnia, dry mouth, and gastrointestinal symptoms [39]. Additionally, it should not be prescribed to patients with seizure disorders, anorexia, bulimia, or opioid use [39]. Naltrexone-bupropion is FDA approved for obesity management. There were three results when searching for PWS and naltrexone–bupropion with one meeting inclusion criteria. This included a 13-year-old with PWS and impulse control on naltrexone–bupropion who had decreased BMI with improved aggression [40].

#### 3.1.8. Other Medications under Investigation

There are other drugs targeting obesity that are being considered as potential therapies in the PWS population. Tesofensine is a triple monoamine reuptake inhibitor of neurotransmitters dopamine, norepinephrine, and serotonin that acts as an appetite suppressant, and has been associated with weight reduction in a placebo-controlled trial of patients with obesity in Denmark [41,42]. In the United States, tesofensine, in combination with metoprolol, has already demonstrated reductions in weight, BMI, and hyperphagia in 18 patients with PWS [40]. Phase 2a trials with tesofensine and metoprolol for the treatment of hypothalamic obesity and PWS have found reductions in weight and hyperphagia scores in adults with PWS [43]. Another drug that is emerging as potentially therapeutic for PWS symptomatology is diazoxide, an ATP-sensitive K+ channel agonist that inhibits insulin secretion from the pancreas and, thus, modulates insulin-sensitive enzymes, leading to suppressed lipogenesis and increased lipolysis. Diazoxide has been shown to decrease fat mass, weight, and blood glucose levels in a mouse model with PWS [44]. There is a pilot trial assessing efficacy and safety of diazoxide choline which found relevant decreases in fat mass and statistically significant reductions in hyperphagia among nine patients with PWS [6]. Larger studies are underway to substantiate the use of diazoxide choline in adolescents and young adults with PWS. Setmelanotide (or RM-493) is an agonist of the appetite-regulating melanocortin-4 receptor which is being investigated for different forms of genetic obesity, including Bardet-Biedl syndrome. A phase 2 trial was completed in adults with PWS and found clinically meaningful weight loss with setmelanotide, though with modest improvements in hyperphagia-related behaviors [43]. Lastly, livoletide is an unacylated ghrelin analogue that has been shown to inhibit the orexigenic effect of unacylated ghrelin in animals, and is hypothesized to have favorable metabolic effects in humans [45]. Unfortunately, phase 2b clinical trials of livoletide in the PWS population did not result in a significant change in hyperphagia and food-related behaviors relative to placebo (ClinicalTrials.gov Identifier: NCT03790865). 

### 3.2. Case Series Results

#### 3.2.1. Participants 

**Figure 1:**
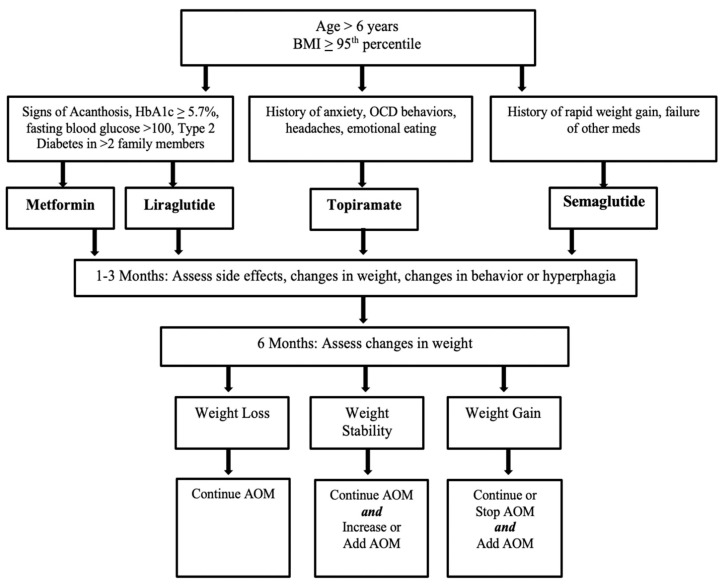
Anti-obesity medication algorithm utilized at our multi-disciplinary PWS center. Side-effect profile, contraindications, comorbidities, and goal mean weight loss are also taken into consideration when collaborating with patients and families in choosing medications. There is continued re-assessment of weight changes, plateaus, and side effects. Abbreviations: OCD, obsessive compulsive disorder; AOM, anti-obesity medication; PWS, Prader-Willi syndrome; BMI, body mass index.

Since the inception of the multi-disciplinary clinic in 2018, 75 patients with PWS have been referred to the PWS program, and 35 have completed the low intensity (12 contact hours/6 months), multi-disciplinary (pediatric endocrinologist, social worker, dietitian, and physical therapist) weight management program. Ten patients were identified who had been recently prescribed AOMs (metformin = 6, topiramate = 5, semaglutide = 2, liraglutide = 3) (Table 2). No patients had been prescribed naltrexone-bupropion. During this time period, no patients were prescribed phentermine, oxytocin, or orlistat. All 10 patients completed a multidisciplinary lifestyle modification program created for patients with PWS and their families. This program was implemented by a pediatric endocrinologist and obesity medicine specialist, registered dietician, and social worker. The patient and one parent attended a 120-min visit monthly for 6 months followed by 30-min maintenance visits occurring every 3 months. At each visit, the patient and parent met with all three providers and created an individualized, goal-based plan for the patient and family to implement over the next month. The interventions focused on creating a food-safe zone, caloric restriction and monitoring, behavioral and psychosocial support, and physical activity. AOMs are prescribed based on a center-specific AOM treatment guideline created from evidence-based prescribing practice by adult and pediatric obesity medicine specialists (Figure 1). Of note, several of the data points reported in this study were collected during the COVID-19 pandemic, which has had significant impacts on daily function, eating structure, and psychosocial support for all of these families, and thereby may have impacted the results reported [46,47].

#### 3.2.2. Metformin 

Six of the ten patients were on full-dose metformin 1000 mg twice daily. All six, completed a 16-week course of metformin. Of those six patients, only one, Case 1, had a decrease in BMI z-score (zBMI) after a four-month course (baseline zBMI: 2.25 SD, zBMI at week 16: 1.9 SD, change: −0.35 SD, weight change: −4.5 kg, concordant height change: +2 cm). During this period, she had an increase of height of 2 cm, correlating with an annualized growth velocity of 5 cm/y. Of note, she remained on the metformin for 24 months and sustained weight loss during that time (−0.93 SD, total weight loss −3.9 kg). Two additional patients maintained their zBMI during the metformin course (Case 6: baseline zBMI: 2.29 SD, zBMI at week 16: 2.31 SD, change: +0.02, weight change: +3.20 kg, concordant height change: +1.1 cm; Case 3: baseline zBMI: 3.1 SD, zBMI at week 16: 3.1 SD, change: 0.0 SD, weight change: +5.1 kg, concordant height change: +3.8 cm). The remaining three cases had an increase in their zBMI (Case 5: baseline zBMI: 2.45 SD, zBMI at week 16: 2.48 SD, change: +0.03 SD, weight change: +5.9 kg, concordant height change: +2 cm; Case 8: baseline zBMI: 3.3 SD, zBMI at week 16: 3.32 SD, change: +0.02 SD, weight change: +0.3 kg, concordant height change: +2.5 cm; Case 10: baseline zBMI: 2.21 SD, zBMI at week 16: 2.23 SD, change: +0.02 SD, weight change: +2.8 kg, concordant height change: +1.7 cm). Overall, parents reported improvement in food-seeking behavior, hyperphagia, appetite, and impulse to eat. There were no side effects reported by any of the parents. 

#### 3.2.3. Topiramate 

Five patients were treated with Topiramate 100 mg nightly for 16-weeks. After a four-month treatment course, two patients had reductions in zBMI (Case 2: baseline zBMI: 2.58 SD, zBMI at week 16: 2.47 SD, change: −0.11 SD, weight change: −1.5 kg, concordant height change: +1 cm; and Case 7: baseline zBMI: 2.64 SD, zBMI at week 16: 2.63 SD, change: −0.01 SD, weight change: +1.0 kg, concordant height change: +0.20 cm). The other three patients showed increased zBMI (Case 4: baseline zBMI: 2.9 SD, zBMI at week 16: 2.95 SD, change: +0.05 SD, weight change: +4.1 kg, concordant height change: +2.7 cm; Case 5: baseline zBMI: 3.1 SD, zBMI at week 16: 3.12 SD, change: +0.02 SD, weight change: +5.5 kg, concordant height change: +1.8 cm; Case 8: baseline zBMI: 2.65 SD, zBMI at week 16: 2.66 SD, change: +0.01 SD, weight change: +1.8 kg, concordant height change: +2.1 cm). Parents of Case 2 reported continued hyperphagia, but no gastrointestinal symptoms, fatigue, or brain fog. Parents of Case 4 reported improvement in behavior, but worsened hyperphagia. Conversely, parents of Case 7 reported decreased appetite and impulse to eat. The only reported side effect was increased sleepiness in Case 5.

#### 3.2.4. Glucagon-Like Peptide-1 Agonist 

##### Liraglutide

Three patients were treated with liraglutide 3 mg daily for 16-weeks. One had decreased zBMI (Case 7: baseline zBMI: 2.43 SD, zBMI at week 16: 2.41 SD, change: −0.02 SD, weight change: −0.1 kg, concordant height change: +1.6 cm), one with no change (Case 9: baseline zBMI: 2.7 SD, zBMI at week 16: 2.7 SD, change: 0.0 SD, weight change: +1.5 kg, concordant height change: +1.3 cm), and one with increased zBMI (Case 10: baseline zBMI: 2.1 SD, zBMI at week 16: 2.12 SD, change: +0.02 SD, weight change: +3.2 kg, concordant height change: +1.1 cm). Liraglutide was the most successful AOM in this case series, given that two out of three patients on liraglutide either maintained or decreased their zBMI. There were no reported side effects.

##### Semaglutide

Two patients were treated with Semaglutide 1 mg weekly for 16-weeks (both combination therapy). One had an increase and one had a decrease in their zBMI at month 4 compared to baseline (Case 5 baseline BMI was 80.4 kg/m^2^, baseline zBMI: 3.31, 12-week monotherapy with topiramate: zBMI 3.29, change: −0.02 SD, weight change: +0.05 kg; topiramate +metformin: starting zBMI: 3.29, at week 12: zBMI 3.26, change: −0.03 SD, weight change: −0.05 kg, concordant height change: +2 cm; topiramate/metformin/semaglutide: starting zBMI: −3.26, at week 12: zBMI 3.24, zBMI change: −0.02SD, weight change: −5.7 kg, concordant height change: +2 cm; Case 6: baseline zBMI: 2.94 SD, zBMI at week 16: 2.96, change: +0.02 SD, weight change: +2.4 kg, concordant height change: +0.1 cm). Case 5, who was on triple therapy with topiramate, metformin, and semaglutide, actually had a significant weight loss during this time of 5.7 kg. Case 6 was on double therapy with metformin and semaglutide. Parents of Case 6 reported worsened hyperphagia during the pandemic. Regarding side effects, Case 5 developed nausea after initiation of semaglutide, which resolved after month 2. There were no other significant side effects.

#### 3.2.5. Combination Treatment 

Based on our single center AOM algorithm, if after 6 months of monotherapy greater than 5% weight loss is not achieved, the recommendation is to trial combination therapy. In this cohort, the following patients completed a trial of a combination of AOMs (Case 5: metformin, topiramate, semaglutide; Case 6: metformin, semaglutide; Case 7: metformin, topiramate, liraglutide; Case 8: metformin, topiramate; and Cases 9 and 10: metformin, liraglutide. Both Case 5 and Case 7 had decreased zBMI with the addition of AOMs—both with the addition of topiramate and liraglutide. No parents reported any significant side effects with addition of another AOM.

## 4. Discussion

Severe obesity remains a life-limiting comorbidity with increasing prevalence in youth with PWS. There remains a paucity of literature on the acceptability, safety, and efficacy of AOMs in this cohort. This study explores the literature on AOM use in youth with PWS and describes cases of initiating AOMs through a single-clinic experience. Consistent with AOM use in pediatric patients with obesity, there is great heterogeneity in response to weight loss medication regimens in the PWS literature [2,12,13]. For all the available treatment options, it appears that there are high responders and low responders to treatment based on psychosocial, pathological, and environmental factors. Our case series demonstrates that patients with PWS and obesity can trial various AOMs with minimal negative effects and variable weight reduction. Further investigation, including large multi-center trials, is needed to better understand the efficacy and safety of AOMs in patients with PWS. Currently, there are several medications under investigation in early phase trials that aim to improve hyperphagia in PWS by directly targeting various specific hypothalamic pathways in an attempt to specific target the hyperphagic signals impacting individuals with PWS [6,7,8]. 

Our literature review for anti-obesity medications in pediatric populations with PWS demonstrates improvement in hyperphagia and social behavior with variable, yet limited effects on BMI or weight loss. Liraglutide, often in combination with other anti-obesity medications, shows the most promising effect on weight loss in this literature review. The articles on liraglutide often included other anti-obesity medications, such as metformin or empagliflozin, with all four demonstrating decreased HbA1c and variable effects on weight without side effects. The three articles on topiramate showed positive changes in mood and hyperphagic behavior, with only one showing statistically significant weight loss. The most common reported side effect in the studies was increased somnolence. Metformin also showed improved hyperphagia without weight loss. The five articles on oxytocin showed inconsistent effects on behavior, which ranged from improved hyperphagia and social behavior to sadness to no effect. There were no changes in BMI or weight. Side effects included nasal irritation and negative effects on behavior, including increased temper outbursts, anger, sadness, and irritability. The case report on naltrexone-bupropion showed decreased BMI and aggression without side effects but results were limited to one patient.

Overall, this review shows significant heterogeneity of weight loss, generally positive behavioral changes in regard to eating behaviors and hyperphagia, and good safety profiles. Our case series shows improved hyperphagia behavior in many, weight loss in several patients, and limited side effects. Overall, more than half of the patients (6) had decreased or unchanged zBMI after AOM initiation. The medication with the greatest percentage of responders was Liraglutide. There were heterogeneous responses for patients on several AOMs. Multiple families reported improved general behavior and decreased hyperphagia with minimal side effects. These data were collected from our multidisciplinary center, where we created an internal protocol to support providers in prescribing AOMs to patients with PWS. The protocol (Figure 1) is designed to be implemented in youth with PWS over the age of six, with obesity or severe obesity. Similar to many adult-prescribing algorithms, the medications are divided into three categories based on mechanism of action and associated patient risk factors. Low starting doses are utilized and then titrated as tolerated to weight-based or adult dosing. The protocol recommends starting with monotherapy and then transitioning to combination therapy if monotherapy is unsuccessful. 

There is limited literature on the use of AOMs in pediatric patients and many of these medications are not FDA-approved for weight management. However, as the evidence base continues to grow around the recommended uses of AOMs in pediatric patients, it becomes critical to design collaborative algorithms to assist providers in harnessing these tools. This would better allow providers to support patients and families as they seek to achieve clinically meaningful weight loss and long-term metabolic health, while decreasing the early onset of obesity-related comorbidities that, for many with PWS, is life-limiting. Further regulatory studies are needed in order to work towards obtaining FDA approval for these medications in younger children, which would subsequently provide families with medication access. This is particularly important given the significant cost of medications and the frequent difficulty obtaining insurance coverage.

Limitations of this literature review include the exclusion of non-English literature. Another limitation is that most articles do not report long-term follow-up, so it is not possible to identify long-term effectiveness or adverse events. Limitations of the case series include only looking at 10 patients with PWS within a wide age range (10–18 years old) as well as variable additional medications (anti-psychotics, hormonal medications) and co-morbidities (diabetes, depression, asthma) that may have affected results. It is also important to note that the initiation of these AOMs be a combination treatment with dietary limitations and increased exercise at home. Furthermore, this study was completed during a global pandemic where there was increased weight gain across many populations in the United States [46,47].

## 5. Conclusions

It is imperative that we find improved treatments for the obesity associated with PWS. Obesity is a leading cause of mortality and high morbidity rates among individuals with PWS, with a 3% annual death rate across all ages [47,48,49]. This contemporary review and case series details 10 cases of youth with PWS on AOMs for weight management and describes how AOMs can be effective at decreasing appetite and reducing BMI for certain patients with PWS. In addition, the side effect profile in this cohort was very mild, and parents reported high satisfaction rates overall with these medications. Further collaborations and studies are required to create a guideline for the use of AOMs in youth with PWS to improve provider familiarity and comfort level with prescribing these medications in this patient population.

## Figures and Tables

**Table 1:** Literature Review and Study Characteristics.

Article	Date	AOMs	Study Design	Sample	Effectiveness	Adverse Events
Smathers et al. [13]	2003	Topiramate	Open Label study	7 individuals Ages: 10–18 years 3 female, 4 male	All with weight loss or reduced weight gain All with improved mood, decreased aggression, less obsessive-compulsive behaviors	3 patients had increased somnolence, all of whom improved with altered dosage or administration timing
East and Maroney [14]	2018	Topiramate	Case Report	11-year-old male	Reduced aggression and “demand for food” following topiramate No impact on BMI	No side effects reported
Consoli et al. [12]	2019	Topiramate	Double-blind, randomized placebo-controlled study—TOPRADER study: 32 placebo 30 topiramate Duration: 8 weeks	62 individuals Ages: 12–45 year 2 female, 30 male	Decreased BMI trend, but without statistical significance Dose-dependent improvement in hyperphagia behavior (Dykens Hyperphagia Questionnaire)	4 patients with sedative effects. 2 patients with infectious episode (bronchiolitis, asthma, sinusitis). Both placebo and topiramate groups had several individuals with biological modifications in hepatic function (3 vs. 4), hyperammonemia (2 vs. 4)
Miller et al. [4]	2014	Metformin	Pilot Study	21 individuals; 6 with early morbid obesity Ages: 7–17 years 11 female, 10 male	Improvement in “food-related distress,” anxiety, ability to be redirected away from food (Hyperphagia Questionnaire) 5 of 13 parents of children with PWS reported children feeling full (often for the first time) No significant weight loss in PWS	7 out of 10 males with PWS reported worsening behavioral problems All of those who stopped metformin had subsequent weight gain
Cyganek et al. [21]	2011	Liraglutide (+Metformin)	Case Report	17-year-old female with diabetes	HbA1c decreased 1.9% and body mass by 3.2 kg over 14 weeks	No hypoglycemia or other side effects
Kim et al. [24]	2020	Liraglutide	Case Report	18-year-old female	Continued previous regimen of metformin, insulin detemir, growth hormone, estrogen Following hospital discharge, was able to maintain BMI with decreased HbA1c while on newly added liraglutide	No reported side effects
Candler et al [25]	2020	Liraglutide + Empagliflozin	Case Report	13-year-old with diabetes	Decrease in HbA1c and glucose on combination of liraglutide + empagliflozin No decrease in glucose or HbA1c while on metformin + insulin or metformin + liraglutide	No reported side effects
Sano et al. [26]	2020	Liraglutide + Empagliflozin	Case Report	19-year-old female with diabetes	With Liraglutide: HbA1c decreased 1.3% after 4 months; No significant change in body weight With addition of empagliflozin, had 7.4% weight loss and 2% decrease in HbA1c	No reported side effects
Einfeld et al. [35]	2014	Oxytocin	Randomized, double-blind, placebo-controlled, crossover trial: 8 weeks of oxytocin, 2-week washout, 8 weeks placebo	30 individuals Ages: 12–30 years 10 female, 20 male	Oxytocin had little impact on any measure	Increase in temper outbursts with higher doses of oxytocin
Kuppens et al. [36]	2016	Oxytocin	Randomized, double-blind, placebo-controlled, crossover trial: Intranasal oxytocin vs. placebo Duration: 4 weeks	25 indiviuduals Ages: 6–14 years 11 female, 14 male	No change in social behavior or hyperphagia in total group In children younger than 11 years, parents reported decreased sadness, anger, conflict, as well as improvement in food-related and social behaviors No significant change in BMI	In children older than 11 years, increased anger and sadness and decreased happiness in oxytocin group No adverse events or other reported side effects
Miller et al. [34]	2017	Oxytocin	double-blind, placebo-controlled crossover study: 5 days of intranasal oxytocin vs. 5 days placebo, followed by 4-week washout	24 individuals Ages: 5–11 years 9 female, 15 male	Decrease in overall anxiety, self-injurious behavior Improvement in socialization, appetite No change in weight	Nasal irritation 4 with increased irritability, resolved
Damen et al. [37]	2021	Oxytocin	Randomized, double-blind, placebo-controlled, crossover trial: Twice daily intranasal oxytocin (dose range 16–40 IU/day) versus placebo Duration: 3 months	26 individuals with PWS Ages: 3–11 years 13 female, 13 male	No significant change in social behavior or hyperphagia were found in total group Oxytocin had positive impact on social and eating behaviors in boys with PWS and children with PWS who had a deletion	No significant side effects
Hollander et al. [38]	2021	Oxytocin	Randomized, double-blind, placebo-controlled trial: 11 oxytocin, 12 placebo Duration: 8 weeks	23 individuals Ages: 5–18 years 5 female, 18 male	Placebo was associated with improvement in hyperphagia and repetitive behaviors; oxytocin was not Oxytocin well-tolerated	Nocturia in individuals given oxytocin
Puri et al. [40]	2016	Naltrexone-Bupropion	Case Report	13-year-old female	Improved eating habits and BMI Decreased aggression	No reported side effects

AOM, anti-obesity medications; BMI, body mass index; PWS, Prader-Willi syndrome.

**Table 2:** Case Series.

Case	Sex	Age (Year)	Genetic Mutation	Other Medical Conditions	Chronic Medications	Anti-Obesity Medication	zBMI Change (≥12 Weeks on AOM)	Side Effect Profile	Behavioral Change (Parental Report)
1	F	12	Uniparental Disomy	Anxiety OCD	Somatropin, coenzyme Q10	Metformin 1000 mg twice daily	−0.35 *	No diarrhea, vomiting, abdominal pain	Improved food-seeking behaviors
2	F	10	Maternal Isodisomy	Asthma Seasonal allergies	Montelukast, fluticasone, cetirizine	Topiramate 100 mg nightly	−0.11 *	No nausea, abdominal pain, fatigue, brain fog	Continued hyperphagia
3	M	10	Deletion of 15q11.2–13	Allergies Insomnia Psychosis	Aripiprazole, guanfacine, cetirizine, fluticasone	Metformin 1000 mg twice daily	0.0 *	No side effects	Continued compulsive aggressive outbursts
4	M	14	Deletion of 15q11.2–13	Hypogonadotropic hypogonadism	Depo-Testosterone	Topiramate 100 mg nightly	+0.05 *	No fatigue or brain fog	Improved behavior, worsened hyperphagia
5	M	12	Uniparental Disomy	Asthma Allergies OSA Hypogonadotropic hypogonadism	Fluticasone albuterol, loratadine	Topiramate 100 mg nightly	−0.02	Increased sleepiness	
					Above medications + tiotropium bromide, montelukast	Metformin 1000 mg twice daily	−0.03 *	No side effects	
					Above medications + Depo-Testosterone, symbicort	Semaglutide 1 mg weekly	−0.02 *	Nausea after semaglutide injections (resolved after 2 months)	
6	M	12	Deletion of 15q11.2–13	Asthma Allergies OSA Hypogonadotropic hypogonadism Anxiety	Flonase, albuterol, loratadine	Metformin 1000 mg twice daily	+0.02	No nausea or vomiting	
					Above medications + Depo-Testosterone, olanzapine, oxcarbazepine, sertraline	Semaglutide 1 mg weekly	+0.02 *	No side effects	Anti-psychotic medications and pandemic have worsened hyperphagia
7	F	17	Deletion of 15q11.2–13	Type 2 diabetes Hypogonadotropic hypogonadism Anxiety OCD	Basaglar, Humalog, Vivelle	Topiramate 100 mg nightly	−0.01 *	No side effects	Decreased appetite and impulse to eat
					Above medications	Liraglutide 3 mg daily	−0.02 *	No side effects	
8	M	15	Deletion of 15q11.2–13	Hypogonadotropic hypogonadism Anxiety	Depo-Testosterone	Metformin 1000 mg twice daily	+0.02	No side effects	Improved hyperphagia but continued weight gain
					Above Medications	Topiramate 100 mg nightly	+0.01	No side effects	Continued weight gain during pandemic
9	F	18	Deletion of 15q11.2–13	Hypertension hypogonadotropic Hypogonadism Type 2 Diabetes	Enalapril, estradiol, Lantus, progesterone	Liraglutide 3 mg daily	0.0 *	No side effects	Hyperphagia worse during pandemic
10	M	12	Deletion of 15q11.2–13	Growth hormone deficiency Asthma Hypogonadotropic hypogonadism	Somatotropin, Albuterol	Metformin 1000 mg twice daily	+0.02	No abdominal pain or nausea	Increased activity
					Above medications + Depo-Testosterone,	Liraglutide 3 mg daily	+0.02 *	No side effects	

* = Medication initiation and weight data collected during COVID-19 pandemic. OCD, obsessive compulsive disorder; OSA, obstructive sleep apnea.

## Data Availability

The datasets from this study will be available from the corresponding author upon written request.
